# Safety and Effectiveness of Integrative Korean Medicine Treatment for Pediatric Patients After Traffic Accidents: Retrospective Chart Review and Survey Research with a Focus on Emotional and Behavioral Symptoms

**DOI:** 10.3390/healthcare13151835

**Published:** 2025-07-28

**Authors:** Yoon Jung Lee, Joo Hee Oh, Dong Jin Jang, Hyo Eun Lee, Ho-Yeon Go, Ju Yeon Kim, Yoon Jae Lee, In-Hyuk Ha

**Affiliations:** 1 Bundang Jaseng Hospital of Korean Medicine, Seongnam-si 13590, Republic of Korea; yoonnung3453@gmail.com (Y.J.L.); christy0123@naver.com (J.H.O.); z65158466@gmail.com (D.J.J.); nunkiii@naver.com (H.E.L.); 2 Korean Internal Medicine, Semyung University, Jecheon-si 38066, Republic of Korea; kohoyeon@gmail.com; 3 Jaseng Spine and Joint Research Institute, Jaseng Medical Foundation, Seoul 06110, Republic of Korea; jyjykim128@jaseng.org (J.Y.K.); goodsmile8119@gmail.com (Y.J.L.)

**Keywords:** integrative Korean medicine treatment, post-accident syndrome, pediatrics, retrospective study

## Abstract

**Background/Objectives**: Providing appropriate treatment for pediatric patients after traffic accidents remains a significant challenge. Furthermore, limited studies have validated the long-term effectiveness and safety of integrative Korean medicine treatment (IKMT) based on follow-up periods of 6 months or longer for pediatric patients. **Methods:** A retrospective chart review was conducted, focused on children aged 0–6 years who visited one of seven Korean medicine hospitals after traffic accident injuries and received IKMT between 1 January 2019 and 30 June 2023. The primary outcome was the Numeric Rating Scale (NRS) scores of chief complaints, and the secondary outcomes were quality of life, adverse events, and satisfaction with IKMT. Statistical analyses were conducted using paired *t*-tests and descriptive statistics, with a significance level of 5%. **Results:** Sixty-four participants were included in the retrospective chart review, and fifty-seven guardians responded to the surveys (mean age: 4.84 ± 1.26 years; mean duration of treatment: 19.20 ± 25.38 days). Among the immediate symptoms after the accidents, flashbacks and intrusive symptoms as well as nightmares and crying were the most common (50.9%). Following treatment, the NRS scores for flashbacks and intrusive symptoms and for nightmares and crying showed meaningful improvements from the time right after the accidents to the survey period. Follow-up confirmed that quality of life scores on all dimensions corresponded with those of healthy children. Nine adverse events were reported, and the participants fully recovered without the need for additional treatment. Furthermore, 91.2% of the survey respondents were satisfied with IKMT. **Conclusions:** IKMT was effective and safe for alleviating the post-accident symptoms in infants and young children aged 0–6 years involved in traffic accidents.

## 1. Introduction

According to the World Health Organization’s Global Status Report on Road Safety [1] published in 2023, there were an estimated 1.19 million road traffic deaths in 2021 alone, and the number of people sustaining nonfatal injuries from traffic accidents (TAs) ranges between 20 and 50 million. According to 2019 statistics for causes of death, road traffic crashes were one of the leading causes of death in children and adolescents aged 5–29 years, ranking 12th among the leading causes of death considering all ages. Furthermore, a study investigating nonfatal injuries in Korean children and adolescents reported that motor vehicle accidents accounted for 11.3% of all injuries, making TAs the third leading cause of injuries [2].

In the current South Korean healthcare system, various Korean medicine (KM) services, such as acupuncture, moxibustion, cupping therapy, Chuna manual therapy, KM physical therapy, and herbal medicine, are provided at KM clinics to improve pain and functional impairment caused by neck pain, lower back pain, and fractures in patients injured in TAs. Furthermore, the Clinical Practice Guideline of Korean Medicine for Traffic Injuries [3] has been developed for KM treatments of whiplash-associated disorders in adult patients. Previous retrospective studies [4,5,6,7] in adult patients have reported symptom improvements and high satisfaction with IKMT. The number of patients visiting KM institutions after TAs has also been increasing. According to automobile insurance statistics from the Health Insurance Review & Assessment Service, medical expenses for KM services have shown a steady increase from approximately KRW 956 billion (USD 697.56 million) in 2019 to KRW 1488 billion (USD 1.089 billion) in 2023 [8].

In the case of pediatric patients who were involved in TAs, there are challenges in providing appropriate diagnosis and treatment because of issues being the lack of self-awareness of symptoms that developed after TAs. In fact, a previous study reported that only approximately 10% of pediatric trauma patients received analgesics in the pre-hospital setting [9]. Moreover, in the case of adult TA patients, their chief complaints primarily consist of musculoskeletal injuries such as sprains, fractures, and dislocations, whereas pediatric patients often experience psychological symptoms [10]. In fact, since children’s ability to adapt to external shock and psychological trauma is less developed, studies have reported that they are more likely to be affected by psychological or neuropsychiatric symptoms such as sleep disorders, anxiety, and panic [11]. A recent study focused on pediatric patients injured in car crashes and who visited a KM hospital also indicated that sleep disturbance was the most common symptom (47.1%), followed by pain at local sites (28.6%) and psychological symptoms such as anxiety, fear, and panic (12.8%) [12], indicating different patterns between adult and pediatric patients.

Among pediatric patients involved in TAs, those with minor trauma not requiring surgery, persistent pain without remarkable abnormal findings on examinations, or psychological symptoms often have limited treatment options within the current conventional Western medicine (WM) services. Owing to these limitations, several pediatric patients visit KM hospitals and clinics for the treatment and management of TA-induced symptoms.

Jo et al. [12] investigated 121 cases of children injured in TAs, reporting the current state of KM treatment for these patients and improvements in their symptoms. Koo et al. [13] reported the effectiveness of inpatient KM treatment in improving the symptoms of 24 pediatric patients with cervical sprains resulting from TAs. Furthermore, Kang et al. [10] reported that KM treatment is effective for addressing night crying and night terror after TAs. Shim et al. [14] reported satisfaction among pediatric patients who were involved in TAs and received KM treatment.

These prior studies involved retrospective analyses of the effectiveness of KM treatment for pediatric patients after TAs or surveys of treatment satisfaction. However, no studies have investigated the long-term effectiveness and safety of KM treatment in terms of symptom relief, functional improvements for returning to daily life, and patient satisfaction following treatment. Furthermore, limited research on TAs has focused on infants and young children who are at the stage of life of experiencing the most rapid physical and mental development.

Addressing these gaps, the current study involved a retrospective analysis of the medical records of infants and young children who visited the Jaseng Hospital of Korean Medicine owing to TA injuries and received integrative KM treatment (IKMT) over the past five years. Additionally, a survey was conducted with their guardians to examine the characteristics of symptoms in pediatric patients as well as the long-term effectiveness, safety, and satisfaction associated with IKMT for treating these symptoms.

Therefore, this study aimed to evaluate the safety and effectiveness of IKMT in children aged 0–6 years who experienced traffic accidents. We hypothesized that IKMT would be associated with improvements in psychological and musculoskeletal symptoms, with a low incidence of adverse events and high caregiver satisfaction.

## 2. Materials and Method

### 2.1. Study Design

This observational study based on a retrospective chart review and prospective long-term follow-up was designed in accordance with the STrengthening the Reporting of OBservational studies in Epidemiology (STROBE) statement. This study was approved by the Institutional Review Board of the Jaseng Hospital of Korean Medicine (file no.: JASENG 2023-11-007; approval date: 20 December 2023), and all the researchers adhered to the Declaration of Helsinki. The study was registered in ClinicalTrials.gov (registration number: NCT06410586).

Regarding the study sample, participant recruitment focused on patients aged 0–6 years who visited any of the seven branches of the Jaseng Hospital of Korean Medicine nationwide (Gangnam, Gwangju, Daejeon, Bucheon, Bundang, Ulsan, and Haeundae) for outpatient and/or inpatient treatments after TA injuries from 1 January 2019 to 30 June 2023; those who met the inclusion/exclusion criteria were selected as eligible participants. We included children aged 0–6 years to encompass both infants and preschool-aged children in Korea.

First, the purpose and content of the study were explained via phone to the guardians of the participants, and an electronic informed consent process was conducted. Two types of consent forms were used: one for the patients’ medical records and the other for consent for the survey of guardians. Medical records and electronic data of the patients were retrospectively obtained if their guardians submitted the consent forms to participate in the study. The prospective survey was conducted using Google Forms. If a guardian did not consent to the use of personal information or participation in the study, the patient was immediately excluded, and the personal information was disposed. If only one of the two consent forms was submitted, the analysis was limited to the data corresponding to the signed consent form received. Medical records were accessed between 5 February 2024 and 8 October 2024 during the consent process and survey.

### 2.2. Participants

#### 2.2.1. Inclusion Criteria

To be eligible for enrollment in the study, the participants had to meet all of the following criteria:

1. Those aged 0–6 years as of the study participation date, who visited the hospital with symptoms caused by TAs, and who received at least one session of IKMT as an inpatient or outpatient;
2. Those whose guardians understood the purpose and content of the study and provided their consent for study participation;
3. Those whose guardians have the communicative ability, as well as the mental and physical capacity, to provide appropriate responses to surveys.

#### 2.2.2. Exclusion Criteria

Patients were excluded from the study if they met the following criteria:

1. Those who received consultation only or WM treatment only at the hospital;
2. Other individuals considered unsuitable for study participation by the researchers;
3. Those with a medical history related to their chief complaint.

### 2.3. Data Collection

For outpatients, data collected from the first to the last visit were used, while for inpatients, data collected during their hospital stay were used. Basic information, such as sex, date of birth, height, weight, major medical history, family history, and any allergies, was collected from electronic medical records (EMRs). Dates of symptom onset, first visit, admission, discharge, treatment period, and findings from radiological examinations were also collected for analysis. Records of objective assessment tools, such as the Numeric Rating Scale (NRS) and EuroQol-5 Dimension-5 Level (EQ-5D-5L) questionnaire, were also collected.

Details of the prescribed KM treatments, including acupuncture, cupping therapy, pharmacopuncture, herbal medicine, and Chuna manual therapy, were collected.

### 2.4. Long-Term Follow-Up Survey

The surveys were structured into three parts based on consensus among the researchers, as follows: changes in symptoms categorized by systems, adverse events (AEs), and treatment satisfaction. In the symptoms section, the severity of pain as well as gastrointestinal, urinary, neurological, and psychological symptoms at the time of the TA and the survey were assessed. Symptoms were collected based on caregiver observation through structured, symptom-based survey items rather than diagnostic terms. Additionally, current quality of life was assessed using the Pediatric Quality of Life Inventory (PedsQL™). In the AEs section, the presence or absence of stomachaches, diarrhea, constipation, itching, and pain at the treatment sites was recorded, along with their timing, if applicable. The satisfaction section was about each KM treatment modality, overall satisfaction with IKMT, and whether the patient was treated at another medical institution, along with the reasons. Additionally, information on growth-related factors, such as current height, weight, gestational age at birth, and birth weight, was also collected.

### 2.5. Intervention

The IKMT provided for the participants consisted of a combination of the following treatment modalities, depending on the condition of each patient:

#### 2.5.1. Acupuncture

For acupuncture therapy, disposable stainless steel filiform needles (0.20 mm × 30 mm, Dongbang Medical, Seongnam, Republic of Korea) or intradermal acupuncture were used. Acupoints were selected based on the pain sites and symptoms. The clinician stimulated the selected acupoints by penetrating the skin with an acupuncture needle. The acupuncture needles were retained for 15 min at a depth of 0.5–1.0 cm, and acupuncture was performed once a day. Intradermal acupuncture was retained for 30 min to 48 h, depending on each clinician’s judgment and the patient’s condition. The total number of acupuncture sessions was determined based on the condition of each patient.

#### 2.5.2. Herbal Medicine

Herbal medicine was prescribed as a ten-day dose for outpatients or upon discharge, to be taken twice daily. For inpatients, it was administered twice daily during hospitalization. The types of herbal medicine were selected based on the clinician’s judgment of the symptoms. The dose was determined based on age as follows: 1/5 of the adult dose for children aged under 6 months, 1/4 of the adult dose for those aged 6 months to 1 year, and 1/2 of the adult dose for those aged 1–7 years.

#### 2.5.3. Other KM Treatments

Cupping therapy was performed on two pain sites for 15 min during the acupuncture sessions, avoiding the areas where acupuncture was administered. Disposable sterile no. 3 cups (Dongbang Medical, Seongnam, Republic of Korea) were used. The number of cupping therapy sessions was determined according to the clinician’s judgment, based on the symptoms of each patient.

### 2.6. Primary Outcome Measures

#### NRS of Chief Complaints

For the NRS, a patient chooses the number that best represents the severity of his or her symptoms on a scale of 0 to 10 (0 indicating no symptoms to 10 indicating the worst pain/symptom imaginable). EMRs were reviewed to collect the NRS scores for the chief complaints at the time of visit or on admission/discharge. Additionally, the surveys collected the NRS scores for pain in each affected area, as well as gastrointestinal, urinary, neurological, and psychological symptoms at the time of the TA and survey.

### 2.7. Secondary Outcome Measures

#### 2.7.1. Quality of Life

PedsQL™ [15] is a metric for assessing health-related quality of life in the pediatric population. Separate questionnaires have been developed according to the age group (≤12 months, 13–24 months, 2–4 years, and 5–7 years) and the respondents (patients themselves or their parents). In this study, the questionnaires for patients were used, and one of the following questionnaires was selected according to the age of the participant concerned: The Parent Report for Infants (ages 13–24 months) of the PedsQL™ 1.0 Infant Scales, The Parent Report for Toddlers (ages 2–4 years) of the PedsQL™ 4.0 Generic Core Scales, and The Child and Parent Report for Young Children (ages 5–7 years) of the PedsQL™ 4.0 Generic Core Scales. The questionnaire for infants consists of five subscales: physical functioning, physical symptoms, emotional functioning, social functioning, and cognitive functioning. The questionnaire for toddlers and young children consists of four subscales: physical, emotional, social, and nursery/daycare functioning. All individual items were assessed on five levels (0, never; 1, almost never; 2, sometimes; 3, often; 4, almost always). These questionnaires were used as part of a prospective survey to assess the quality of life of pediatric patients after IKMT. Due to the use of different validated versions of the questionnaire, it was not intended as a subgroup analysis.

The EQ-5D-5L questionnaire [16] is used to assess five dimensions of a patient’s quality of life: mobility, self-care, usual activities, pain/discomfort, and anxiety/depression. Each dimension is rated on five levels: “no problems,” “slight problems,” “moderate problems,” “severe problems,” and “extreme problems.” In this study, EQ-5D-5L scores were calculated using a weighted model with values estimated for the Korean population. The EQ-5D-5L scores were retrospectively collected from EMRs.

#### 2.7.2. AEs

The AEs previously reported in KM treatment studies [17,18] and those considered likely to occur were predefined through consensus among the research team. The survey was then conducted to assess the occurrence of these AEs, along with any treatments provided and the patients’ current condition.

#### 2.7.3. Patient Satisfaction

Satisfaction with KM treatment was evaluated through questions addressing the reasons for choosing KM institutions, their satisfaction levels with IKMT, and whether and why they sought care at other medical institutions after IKMT.

### 2.8. Statistical Analysis

All statistical analyses were performed using SAS ver. 9.4 (SAS Institute, Inc., Cary, NC, USA). Continuous variables were summarized using means and standard deviations, and categorical variables were represented using frequencies and percentages. All statistical analyses were performed based on two-tailed tests, and the significance level was set at 5%.

To evaluate IKMT effectiveness, the changes in NRS scores immediately after the TA and at the time of the survey were analyzed using a paired *t*-test with a 95% confidence interval. The Wilcoxon signed-rank test was performed when the assumption of normality was not met, based on normality testing. Missing data were handled using available case analysis. In the long-term follow-up survey, the occurrence of AEs, reasons for satisfaction or dissatisfaction with KM treatment, overall satisfaction, and status of receiving additional treatment after IKMT were reported in terms of the number of patients and percentages.

## 3. Results

**Figure 1:**
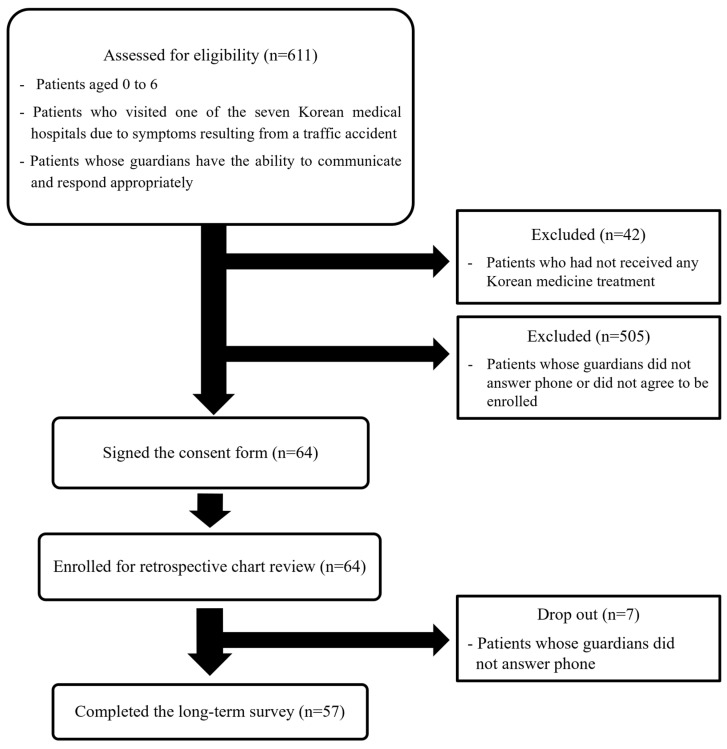
Patient flow of this study.

Among the patients aged 0–6 years who visited the Jaseng Hospital of Korean Medicine (Gangnam, Gwangju, Daejeon, Bucheon, Bundang, Ulsan, and Haeundae) for outpatient or inpatient treatment after a TA injury from 1 January 2019 to 30 June 2023, a total of 611 patients were considered eligible according to the inclusion/exclusion criteria. After excluding 42 patients who did not receive KM treatment and 505 patients whose guardians did not answer phone or disagree to be enrolled, 569 were eligible for the survey. Of these, the guardians of 64 patients provided their consent for the use of EMR data and survey participation. Finally, a total of 57 respondents completed the questionnaires (Figure 1). The surveys were conducted from 5 February 2024 to 8 October 2024. For variables from EMR, only patients with available data were included in the analysis, and the number of patients was reported accordingly for each outcome.

### 3.1. Basic Characteristics

The mean age of the participants was 4.84 ± 1.26 years, of which 38 (59.4%) were boys and 26 (40.6%) were girls. Of the 64 participants, 1 received inpatient treatment, and the other 63 received outpatient treatment. The average duration from the date of onset to the first visit was 4.64 ± 11.29 days, ranging from 0 days to 84 days. For inpatients, the time from onset to admission was two days, and the length of hospital stay was five days. The mean total duration of outpatient treatment was 19.20 ± 25.38 days, ranging from 1 day to 143 days.

None of the participants reported any major medical history, including atopic dermatitis; diseases of the respiratory, cardiovascular, neurological, or gastrointestinal systems; developmental disorders; surgical history; or fracture.

The mean height of the participants was 112.65 ± 9.72 cm, and the mean weight was 20.02 ± 4.30 kg. The mean gestational age at the time of delivery was 38.49 ± 2.05 weeks, and the mean birth weight was 3.21 ± 0.48 kg. Regarding the type of delivery, 27 respondents (47.4%) reported vaginal delivery, while 30 (52.6%) reported cesarean section (Table 1).

### 3.2. Treatment Details

Twenty-nine participants (45.3%) received acupuncture, with an average of 4.62 ± 5.25 sessions (range: 1–27). Cupping therapy was performed for four participants (6.2%), with an average of 2.50 ± 2.38 sessions. Herbal medicine was the most commonly administered KM treatment modality (53 participants; 82.8%), with an average of 1.43 ± 0.80 prescriptions.

### 3.3. Symptom Characteristics

#### Symptom Characteristics Investigated at the Initial Visit

According to EMR data, neck pain was the most common chief complaint at the first visit (20 participants), followed by psychological symptoms (13 participants) and lower back pain (11 participants). The mean NRS scores for neck pain, psychological symptoms, and lower back pain assessed at the first visit were 3.25 ± 1.52, 3.69 ± 1.70, and 3.27 ± 1.79, respectively. Additionally, three participants complained of pain at other sites, such as the shoulder and face, with a mean NRS score of 4.00 ± 1.00. Participants who received inpatient care complained of neck and shoulder pain, with an NRS score of 7.00 (Table 2).

### 3.4. Symptom Characteristics Investigated from the Survey

The survey data revealed that the most frequently reported symptoms following the TA, in descending order, were psychological, gastrointestinal, musculoskeletal, neurological, and urinary symptoms. Among psychological symptoms, “flashbacks and intrusive symptoms” and “nightmares and crying” were reported by 29 participants (50.9%), respectively, followed by “sleep disturbances” in 25 participants (45.6%) and “anxiety and avoidance” in 22 participants (38.6%). Among the gastrointestinal symptoms, anorexia was reported by 25 (43.9%) participants and nausea and vomiting by 15 participants (26.3%). Regarding musculoskeletal pain as the chief complaint, neck pain was the most common, reported by 20 participants (35.1%), followed by lower back pain in 11 participants (19.3%) and lower and upper limb pain in 9 participants (15.8%). Regarding neurological symptoms, headache was reported by 14 participants (24.6%) and dizziness by 11 participants (19.3%). As for urinary symptoms, nocturnal enuresis was reported by 10 participants (17.5%) and frequent urination by 8 participants (14%) (Table 3).

### 3.5. NRS of Chief Complaints

**Figure 2:**
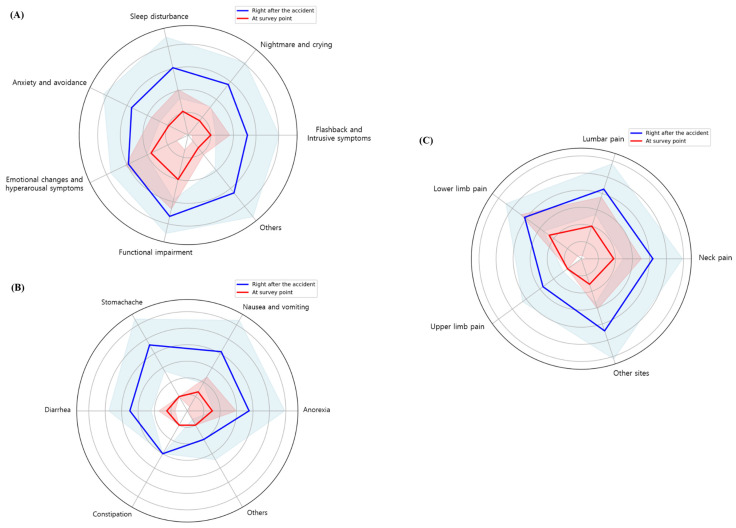
Changes in numerical rating scale (NRS) scores for psychological and digestive symptoms and musculoskeletal pain are investigated using the survey data. (**A**) Psychological symptoms; (**B**) digestive symptoms; (**C**) musculoskeletal pain. The NRS score at the time of the accident is marked in blue, while the NRS score at the time of the survey is marked in red. The shaded area represents the 95% confidence interval.

Changes in the NRS scores for each symptom were calculated based on the difference between the time of the accident and the time of the survey (Table 3 and Figure 2).

#### 3.5.1. NRS of Psychological Symptoms

The mean NRS score for flashbacks and intrusive symptoms reported by 29 participants (50.9%) at the time of the TA was 5.24 ± 2.80, which decreased to 2.00 ± 1.67 at the time of the survey, showing a reduction of 3.24 ± 2.5 (*p* < 0.001). Similarly, the mean NRS score for nightmares and crying decreased from 5.69 ± 2.45 at the time of the TA to 1.62 ± 1.50 at the time of the survey, a reduction of 4.07 ± 2.27 (*p* < 0.001). The NRS scores for other psychological symptoms, including sleep disturbances (*p* < 0.001), anxiety and avoidance (*p* < 0.001), emotional changes and hyperarousal (*p* = 0.001), and others (*p* < 0.001), also showed meaningful improvements compared to those reported at the time of the TA. However, no significant change was observed in the scores for functional impairment (*p* = 0.300) (Figure 2A).

#### 3.5.2. NRS of Gastrointestinal Symptoms

Among the post-accident gastrointestinal symptoms, anorexia, nausea, and headache were the most frequently reported. For anorexia, the mean NRS score was 3.76 ± 2.17 at the time of the TA, which reduced to 1.52 ± 1.45 at the time of the survey, a decrease of 2.24 ± 1.96 (*p* < 0.001). The mean NRS score for nausea and headache decreased from 4.13 ± 2.20 at the time of the TA to 1.33 ± 1.05 at the time of the survey, a reduction of 2.80 ± 2.31 (*p* < 0.001). The mean NRS score for stomachache also showed a meaningful improvement (*p* = 0.011), whereas the score for diarrhea did not show any significant change (*p* = 0.078) (Figure 2B).

#### 3.5.3. NRS of Musculoskeletal Pain

The score for neck pain decreased meaningfully from 4.20 ± 1.74 at the time of the TA to 1.90 ± 1.62 at the time of the survey, reflecting a reduction of 2.30 ± 2.08 (*p* < 0.001). Similarly, the NRS score for lower back pain decreased from 4.27 ± 1.56 to 2.00 ± 1.79, a reduction of 2.27 ± 2.05 (*p* = 0.004). For range of motion (ROM), six (30%) and three (27.3%) participants showed limitations in the neck and lower back, respectively, at the time of the TA; however, all of them had recovered by the time of the survey.

The NRS score for lower limb pain also decreased meaningfully, from 4.11 ± 1.36 at the time of the TA to 1.78 ± 1.30 at the time of the survey, a reduction of 2.33 ± 2.06 (*p* = 0.003), though one patient reported limited ROM at both time points. The NRS score for upper limb pain improved meaningfully, decreasing from 2.78 ± 1.48 at the time of the TA to 1.78 ± 1.48 at the time of the survey, a reduction of 1.00 ± 0.00 (*p* = 0.007). Meaningful improvements were also observed in the NRS scores for pain at other sites (*p* = 0.005) (Figure 2B).

#### 3.5.4. NRS of Neurologic Symptoms

The NRS score for headache decreased from 4.00 ± 1.57 at the time of the TA to 1.00 ± 0.00 at the time of the survey, reflecting a reduction of 3.00 ± 1.57 (*p* < 0.001). The NRS score for dizziness decreased meaningfully from 3.27 ± 1.74 at the time of the TA to 1.36 ± 0.92 at the time of the survey, a reduction of 1.91 ± 1.81 (*p* = 0.006).

#### 3.5.5. NRS of Urinary Symptoms

The mean NRS for nocturnal enuresis, which was reported in most pediatric participants, was 3.60 ± 1.58 at the time of the TA, and this score reduced to 1.60 ± 1.07 at the time of the survey, showing a meaningful decrease of 2.00 ± 1.49 (*p* = 0.002). In case of frequent urination, the NRS score decreased from 3.88 ± 2.17 at the time of the TA to 1.12 ± 0.35 at the time of the survey, a decrease of 2.75 ± 1.91 (*p* = 0.005). No significant changes in diurnal enuresis scores were observed (*p* = 0.186).

### 3.6. Quality of Life

#### 3.6.1. PedsQL™

In the 5–6-year age group (N = 41), the oldest age group, the PedsQL™ scores for physical, emotional, social, and school functioning were 98.48 ± 3.90, 94.27 ± 8.84, 99.02 ± 3.91, and 98.54 ± 3.40, respectively. In the 2–4-year age group (N = 15), the scores for physical, emotional, social, and school functioning were 95.83 ± 8.73, 87.00 ± 14.49, 94.67 ± 10.60, and 95.00 ± 8.21, respectively. Table 4 presents the PedsQL™ scores for each dimension and the summary scores for all age groups (Table 4).

#### 3.6.2. EQ-5D-5L

The participant who received inpatient treatment had a record of the EQ-5D-5L score in the EMR. For this patient, the EQ-5D-5L score improved from 0.841 at admission to 0.91 at discharge.

### 3.7. AEs

The reported AEs included three cases of diarrhea or soft stools (5.3%) and three cases of nocturnal enuresis (5.3%), followed by one case (1.8%) each of constipation or tenesmus, itching at the treatment site, and pain at the treatment site. Except for one case, all AEs were reported to have occurred immediately after treatment. All nine AE cases were followed up without specific treatment, and all symptoms of AEs were reported to have resolved by the time of the survey.

### 3.8. Satisfaction

Twenty-one respondents (36.8%) described their reasons for choosing KM hospitals to treat the post-accident symptoms of their children. The most commonly cited reason (by seven respondents) was the belief that, given the young age of the patients, it would be challenging to perform examinations or treatments in WM institutions.

Regarding overall satisfaction with IKMT, participants’ evaluations were as follows: 29 (50.9%), “very satisfied”; 23 (40.4%), “satisfied”; 4 (7%), “average”; 1 (1.8%), “dissatisfied.” Regarding satisfaction with the specific treatment modalities, 22 of 26 participants (84.6%) who received acupuncture, 27 of 41 participants (65.9%) who received herbal medicine, and all 3 participants (100%) who received cupping therapy expressed satisfaction with the respective treatment. The most frequently cited reason for satisfaction with acupuncture and herbal medicine was reduced anxiety and psychological symptoms, followed by pain relief. None of the participants reported dissatisfaction with acupuncture therapy or cupping therapy. However, all four participants (7%) who expressed dissatisfaction with the herbal medicine treatment cited the unpleasant taste of the herbal medicine as the reason (Supplementary Table S2).

Three participants (5.3%) received KM treatment at another medical institution after completing IKMT. This included one patient receiving KM treatment only from another institution, one patient receiving WM treatment, and one patient receiving both KM and WM treatment. Regarding their reasons for visiting another medical institution, one respondent stated that they sought other types of treatment not provided by KM hospitals because of limited symptom improvements, whereas the other two cited personal reasons. Two respondents noted additional symptom improvements after receiving treatment at another medical institution, whereas one reported that the symptoms remained similar to those after IKMT.

## 4. Discussion

This study involved a retrospective chart review and a prospective observational study of infants and young children aged 0–6 years who visited one of seven branches of the Jaseng Hospital of Korean Medicine (Gangnam, Gwangju, Daejeon, Bucheon, Bundang, Ulsan, and Haeundae) following TA injuries. These patients received IKMT as inpatients or outpatients between 1 January 2019 and 30 June 2023. The effectiveness of KM treatment and treatment satisfaction were analyzed.

The mean age of the 64 participants was 4.84 ± 1.26 years, with 38 (59.4%) boys and 26 (40.6%) girls: a slightly higher proportion of boys. None of the participants had significant medical histories. Based on their mean age, the average height and weight were within the 95th and 85th percentiles [19], respectively, confirming that the study included healthy infants and young children. Of these participants, 63 received outpatient treatment, and 1 received inpatient treatment. It could be attributed to the absence of pediatric wards at the included institutions. The average duration of treatment was 19.20 ± 25.38 days, with a range of 1–143 days. Additionally, the trauma severity of the included patients was presumed to correspond to NACA levels I–II [20], indicating minor to moderate injuries manageable in outpatient settings.

According to the EMRs, the most frequently recorded chief complaints at the initial visit were neck pain, psychological symptoms, and lower back pain. In contrast, the survey indicated that the most frequently reported symptoms were psychological, gastrointestinal, musculoskeletal, neurological, and urinary symptoms, in that order. Furthermore, the mean NRS score for neck pain was 3.25 ± 1.52, as recorded in the EMR at the first visit, compared to 4.20 ± 1.74, as reported in the survey. These findings suggest discrepancies in the types and severity of chief complaints recorded in the EMR at the initial visit versus those reported in the survey. The possible reasons are summarized below.

First, the period from the date of onset to the first visit was 4.64 ± 11.29 days on average, which may have led to differences in the NRS scores at the TA and those at the initial visit. Second, the survey responses relied on respondents’ memories, which may have contributed to differences compared with the EMRs. For example, neck pain may have a delayed onset, as in cases of soft tissue strain, which may have emerged or intensified several days later. Third, for pediatric patients, more symptoms may be observed at home over time. For example, psychological symptoms, such as fear of getting into a car, or urinary symptoms often manifest in daily life and may not have been mentioned during the initial visit. Additionally, at the initial visit, symptoms are typically assessed through open-ended questions in general clinical practice. Given that adults generally visit KM institutions for the treatment of musculoskeletal pain after TAs, the guardians may have primarily described pain-related symptoms rather than other types of symptoms, such as psychological, gastrointestinal, or urinary issues. Meanwhile, the follow-up survey used multiple-choice questions across various systems, likely resulting in capturing a broader range of symptoms.

After the TA, among the symptoms that occurred in the various systems (e.g., psychological, musculoskeletal, gastrointestinal, urinary, and nervous systems), psychological symptoms were reported with the highest frequency. This pattern differed from adult patients who received KM treatment after TAs, mostly reporting musculoskeletal pains in neck and lower back, as well as headache and dizziness [21]. In addition, the NRS scores for psychological symptoms were the highest. Symptoms associated with acute stress reactions, such as intrusive symptoms, night crying, sleep disturbances, anxiety, hyperarousal, and functional impairment, were examined. Among these, the majority of participants (50.9%) reported experiencing “flashbacks and intrusive symptoms” and “nightmares and crying.” Furthermore, symptoms such as “sleep disturbances” (45.6%), “anxiety and avoidance” (38.6%), and “emotional changes and hyperarousal” (24.6%) were also shown to occur at higher rates than symptoms in other systems.

Previous studies have reported that road TAs in children often lead to psychological and physical/somatic symptoms, which can result in long-term, chronic consequences such as post-traumatic stress disorder (PTSD) [22]. Furthermore, even minor injuries from road TAs can cause significant psychological distress, potentially leading to PTSD in children [23]. Dai et al. [24] reported a PTSD prevalence rate of approximately 20% in children and adolescents following road TAs. Olofsson et al. [25] reported that approximately 30% of children and adolescents develop PTSD within one month after an accident, with 13% continuing after three to six months.

In this study, more than 50% of pediatric patients who visited KM hospitals after TAs exhibited symptoms consistent with acute stress reactions. These findings highlight the urgent need for discussions on the role of KM interventions in the early stages of stress reactions to prevent the development of chronic PTSD in children.

In addition to psychological symptoms, anorexia and nausea were reported in high percentages. One proposed mechanism involves activation of the emetic center by signals from the limbic system, which is associated with experience and emotion. This mechanism explains how nausea and vomiting can be triggered by stress or fear [26].

In this study, neck pain was the most frequently reported musculoskeletal symptom. According to previous studies, the cervical spine is the most commonly injured region of the spine in TAs among children under 8 years of age, with cervical spine fractures accounting for approximately 20–25% of all spine fracture cases. A prior study reported that cervical spine injuries were observed in 176 out of 6065 child deaths caused by TAs [27]. Furthermore, the head and cervical spine have been identified to sustain the most injuries, resulting in permanent medical impairment (PMI) in child injured in car crashes. The risk of PMI was 3% for cervical spine injuries [28]. Whiplash, a common injury in motor vehicle accidents, is known to cause headaches as well as cervical spine pain. Previous research has shown that 82% of patients who experience neck pain after an injury also experience headache [29]. These results align with the findings of this study, in which headache was the most frequently reported among the nervous system complaints. Meanwhile, extremity injuries have been reported as the most common in emergency department-based pediatric trauma studies [30], and head- and neck-related complaints may be more frequently observed in outpatient settings, as seen in our sample.

The duration from the date of onset to the first visit varied widely, ranging from 0 to 84 days, which may have influenced the NRS scores recorded at the first visit. Consequently, the effectiveness of IKMT was evaluated based on the NRS scores reported at the time of the TA and those collected during the survey.

According to the NRS scores reported in the survey, all types of musculoskeletal pain, including neck and lower back pain, showed meaningful improvements compared with the severity at the time of the TA. Furthermore, anorexia, nausea, vomiting, stomachache, nocturnal enuresis, frequent urination, headache, and dizziness showed meaningful improvements. Most psychological symptoms showed meaningful improvements as well. In the comparison of right after the TA and present NRS scores from the survey, all symptom categories except for other digestive symptoms showed a large effect size, with Cohen’s *d* values of 0.8 or greater.

Among the different KM treatment modalities, herbal medicine (82.8%), acupuncture (45.3%), and cupping therapy (6.2%) were the most commonly received treatments by pediatric patients. For the herbal medicine, the clinicians selected formulas commonly prescribed for post-accident symptoms in adults [31,32], for pain relief and alleviation of psychological symptoms. A previous study reported that the group receiving IKMT including acupuncture, cupping therapy, and herbal medicine for four weeks (HM group) showed significant improvements in overall symptoms—such as musculoskeletal, neurological, and psychological symptoms—compared with the group that received IKMT without herbal medicine for the same period (control group). Additionally, the HM group experienced a shorter recovery time, with NRS scores reduced by more than 50%, compared with the control group [31]. Xiang et al. [33] performed a meta-analysis and reported that acupuncture resulted in significant pain relief compared with sham acupuncture and analgesic injections in adult patients. Moreover, the pain-relieving effects of acupuncture were more long lasting than those of analgesics. Consistent with the previous research, in this study, IKMT demonstrated notable improvements in psychological, musculoskeletal, and neurological symptoms, which were the most frequently reported symptoms following TAs.

Studies have shown that road TAs in infants and young children significantly affect their growth, development, and quality of life. Landolt et al. [34] reported that PTSD symptoms in pediatric patients one month after a TA showed significant correlations with the quality of life at one month and one year, serving as a significant predictor of quality of life one year after a TA. Arnberg et al. [35] found that pediatric patients who were directly affected in a car crash had a higher prevalence of PTSD even 20 years after the accident. Accordingly, this study also evaluated the quality of life of pediatric patients during follow-up.

In this study, PedsQL™ was used to monitor the quality of life after TAs. The results showed that in the 5–7-year-old and 2–4-year-old groups, all the scores across dimensions (physical, emotional, social, and school functioning) and the summary scores for physical and psychosocial health were better than the average scores of healthy children without special healthcare needs, as reported in a previous study; for healthy children under 8, the total score was 83.7 ± 15.2 [36]. One participant in the 13–24-month group had an emotional functioning score comparable with that of children with chronic illnesses [37]. For this participant, the guardian reported NRS scores of over 7 for the four psychological symptoms (nightmares and crying, sleep disturbances, anxiety and avoidance, and functional impairment) at the time of the TA. Furthermore, the time from the TA to the first visit was relatively long (21 days). This case highlights the significance of early intervention in pediatric patients with severe psychological symptoms. However, owing to the limited number of cases in this study, further research is needed to examine the differences in symptom improvement with and without early intervention.

The AEs reported in this study included three cases of diarrhea or soft stools, three cases of nocturnal enuresis, and one each of constipation, itching, and pain, totaling nine cases involving six patients. The diarrhea or soft stools were considered possibly related to the treatment; nocturnal enuresis and constipation or tenesmus were assessed as unlikely related; itching at the treatment site was probably related; and pain at the treatment site was possibly related. Evaluated retrospectively by researchers, the causal relationships should be interpreted with caution. All six patients had received herbal medicine treatment, and acupuncture was also performed for four of them. Jung et al. [17] reported AEs in 9 out of 212 children (4.2%) who took herbal medicine for more than one week, with most AEs being gastrointestinal symptoms such as stomachache (five cases), diarrhea (three cases), constipation (one case), tenesmus (one case), soft stool (one case), and vomiting (one case). Lee et al. [18] also found that among the AEs reported in 159 pediatric patients who took herbal medicine, gastrointestinal disorders accounted for the highest proportion (70%), with diarrhea (25%) and stomachache (20%) showing high frequencies. Both studies noted that the onset of AEs was primarily within three days of starting herbal medicines, with gastrointestinal symptoms often developing within a few hours and resolving shortly thereafter. In this study, gastrointestinal symptoms were the most frequently reported, with nearly all symptoms occurring immediately after treatment and resolving over time, reflecting a pattern similar to the findings of previous studies. In children, because of the immature digestive system, increased water intake by herbal medicine, and sensitivity to unfamiliar substances, gastrointestinal symptoms may have frequently occurred. These findings suggest that such AEs are predictable and manageable. Therefore, clinicians may use herbal medicine, considering the child’s constitution and digestive condition, and make appropriate modifications to the herbal formula.

In this study, acupuncture, herbal medicine, and cupping therapy were administered to pediatric patients involved in TAs. Most patients who received cupping therapy and acupuncture (100% and 84.6%, respectively) reported satisfaction with the treatment. Meanwhile, 65.9% of the participants expressed satisfaction with the herbal medicine treatment, with all dissatisfied respondents citing the taste of the herbal medicine as the reason for their dissatisfaction. A previous survey study on KM treatment satisfaction among pediatric patients involved in TAs [14] also reported that 31.6% and 24.2% of the respondents selected cupping therapy and acupuncture, respectively, as the most satisfactory KM treatments. However, 42.1% of the respondents chose herbal medicine as the most difficult to comply with, with 20% attributing this to the bitter taste. These findings align with the results of this study, where a high percentage of patients expressed satisfaction with acupuncture and cupping therapy, while several noted dissatisfaction with the taste of herbal medicine. Therefore, there is a need to develop and introduce herbal medicine formulations that are easier for infants and young children to intake. As for Chuna manual therapy, it is a representative KM treatment widely used for various conditions, such as musculoskeletal pain [38]. Previous studies with pediatric patients involved in TAs [13,14] reported that fascia Chuna manual therapy was administered to most participants. However, none of the participants in this study received Chuna manual therapy. Since the data were from multiple institutions, it was difficult to accurately determine the reason for applying Chuna manual therapy, but the limited use likely reflects clinical judgment by each clinician, regarding its feasibility in young children. Further research is needed to determine whether this absence was owing to the participants’ age, the clinical judgment of the Korean Medical Doctors (KMDs), treatment settings, or other reasons.

This study has a few limitations that must be noted. First, the participants were children aged 0–6 years who required their guardians to complete the survey on their behalf. In the EMR as well, age-appropriate validated tools were often not used, and the NRS was recorded based on caregivers’ reporting and the children’s expression. Younger pediatric patients have limited articulation and communication abilities, and the survey responses, provided by their guardians, may not accurately reflect the children’s intentions. In fact, a prior study on post-traumatic stress reactions in children after TAs that involved separate interviews with children and their parents [30] reported that children exhibited significantly more post-traumatic stress reactions four weeks after the TAs than what was perceived by their parents, with the discrepancy increasing in younger children. Additionally, it should be noted that significant discrepancies have been reported between child and parent reports on PedsQL™ subscales [39]. Since parents tend to rate their child’s quality of life more positively, the current quality of life may have been overestimated, particularly for emotional symptoms that are less visible. Second, because most pediatric patients were in the same vehicle as their guardians during the TA, they naturally received treatment together. Thus, the guardians’ perspectives may have influenced the survey responses, making it challenging to separate the guardians’ views. Third, although the study included children aged 0–6 years, most participants were between 2 and 6 years old. Thus, this age range may limit the applicability of our findings to infants and should be interpreted accordingly. Fourth, although the intensity of pain was well documented at the initial visit, it was irregularly recorded during subsequent visits. This limitation made it challenging to accurately assess progress. Fifth, the causality and severity of AEs were assessed during the surveys rather than at the time of their occurrence, resulting in a lack of specific details and grading regarding the AEs. Additionally, similar symptoms may have been reported as either AEs or post-accident symptoms, which could have led to misclassification. Some symptoms, such as nocturnal enuresis, may reflect psychological distress from the accident rather than treatment-related AEs. Fifth, considering the low response rate, participants may have been biased toward those with greater trust or satisfaction with the hospital. Sixth, this study did not adjust for potential confounding variables such as injury severity, symptom duration, number of symptoms, and concurrent treatments. As a result, the effectiveness of IKMT may have been underestimated or overestimated. For example, greater treatment effects might have been observed in patients with mild injuries, shorter symptom durations, fewer symptoms, or those who received other concurrent treatments. Lastly, this was an observational, single-arm study, making it impossible to compare the effectiveness of IKMT for the participants with a control group.

Based on the results of this study, we propose the following recommendations for future clinical practice: First, nonverbal pain scales, such as the Faces Pain Rating Scale, should be used during each hospital visit, and more systematic interviews should be conducted to assess the various symptoms for pediatric patients. Second, several respondents indicated that they chose KM institutions because they believed that WM institutions might face challenges in performing tests or providing active treatments for young children. We may refer to the KM clinical practice guidelines [3] for adults with traffic injuries, which recommend acupuncture for neck pain (Grade B), herbal medicine for neck and low back pain (Grade C), and IKMT for PTSD and acute stress reactions (Grade C). Given the significant impact of TAs on the growth and emotional development of infants and young children, KM treatment could be considered to provide a wide range of treatment options for patients and their guardians. Third, close monitoring of and careful attention to psychological symptoms, neck pain, and anorexia are required, as these are frequently reported during the treatment of pediatric patients involved in TAs. Acupuncture therapy could be considered based on the patient’s condition, and strategies to improve herbal medicine compliance should be developed to ensure effective treatment. Fourth, infants and young children should be closely monitored for gastrointestinal symptoms, such as diarrhea or soft stools, following herbal medicine treatment. Finally, prospective comparative studies with age-appropriate, validated measures are needed to more clearly assess the clinical effectiveness of IKMT. Additionally, prospective cohort studies or matched control designs are warranted to better control for confounding factors.

## 5. Conclusions

This study used a retrospective chart review and a prospective survey to examine treatment effectiveness, long-term treatment effects, safety, and satisfaction associated with IKMT for TA injuries in pediatric patients aged 0–6 years who received inpatient or outpatient treatment at KM hospitals. Despite some limitations, the findings of this study are expected to provide preliminary insights for clinicians in managing pediatric patients involved in TAs in the future.

## Figures and Tables

**Table 1:** Basic characteristics of the included participants.

Variables		N (%)	Mean ± SD
Sex			
	Male	38 (59.4)	
	Female	26 (40.6)	
Age (years) *			4.84 ± 1.26
Age distribution (years)	1	1 (1.6)	
	2	2 (3.1)	
	3	8 (12.5)	
	4	10 (15.6)	
	5	17 (26.6)	
	6	26 (40.6)	
Care type			
	Outpatient	63 (98.4)	
	Inpatient	1 (1.6)	
Significant medical history		0 (0)	
Time from date of onset–date of first visit (days)			4.64 ± 11.29
Time from date of onset–date of admission (days)			2.00 ± NA
Total duration of treatment (days)			19.20 ± 25.38
Current height (kg)			112.65 ± 9.72
Current weight (cm)			20.02 ± 4.30
Delivery type			
	Vaginal delivery	27 (47.4)	
	C-section	30 (52.6)	
Birth weight (kg)			3.21 ± 0.48
Gestational age (weeks)			38.49 ± 2.05

* Median [IQR] for age = 5 [4–6]; NA: Not available.

**Table 2:** Symptom characteristics noted in electronic medical records at the initial visit.

Numerical Rating Scale	N	Mean ± SD
Symptoms of outpatients at the initial visit		
Neck pain	20	3.25 ± 1.52
Lower back pain	11	3.27 ± 1.79
Other symptoms	3	4.00 ± 1.00
Psychological symptoms	13	3.69 ± 1.70
Symptoms of inpatients on the day of admission		
Neck pain	1	7.00 ± NA
Shoulder pain	1	7.00 ± NA

NA: Not available.

**Table 3:** Symptom changes investigated from a long-term follow-up survey.

	N (%)	NRS ** (Mean ± SD)			*p*-Value *	Cohen’s d
		Right After the TA	Present (At the Survey)	Changes		
**Psychological symptoms**						
Flashbacks and intrusive symptoms	29 (50.9)	5.24 ± 2.80	2.00 ± 1.67	3.24 ± 2.59	<0.001	1.25
Nightmares and crying	29 (50.9)	5.69 ± 2.45	1.62 ± 1.50	4.07 ± 2.27	<0.001	1.79
Sleep disturbances	26 (45.6)	6.08 ± 2.76	2.15 ± 1.97	3.92 ± 2.77	<0.001	1.42
Anxiety and avoidance	22 (38.6)	5.55 ± 2.76	1.91 ± 1.69	3.64 ± 2.74	<0.001 †	1.33
Emotional changes and hyperarousal	14 (24.6)	5.86 ± 1.75	3.64 ± 2.50	2.21 ± 2.01	0.001	1.10
Functional impairment	3 (5.3)	7.33 ± 1.53	4.00 ± 2.65	3.33 ± 4.16	0.300	0.80
Others	14 (24.6)	6.50 ± 2.68	1.43 ± 0.76	5.07 ± 2.70	<0.001	1.88
**Digestive symptoms (gastrointestinal system)**						
Anorexia	25 (43.9)	3.76 ± 2.17	1.52 ± 1.45	2.24 ± 1.96	<0.001 †	1.14
Nausea, vomiting	15 (26.3)	4.13 ± 2.20	1.33 ± 1.05	2.80 ± 2.31	<0.001 †	1.21
Stomachache	5 (8.8)	4.60 ± 1.82	1.00 ± 0.00	3.60 ± 1.82	0.011	1.98
Diarrhea	4 (7.0)	3.50 ± 1.29	1.25 ± 0.50	2.25 ± 1.71	0.078	1.32
Constipation	2 (3.5)	3.00 ± 0.00	1.00 ± 0.00	2.00 ± 0.00		
Others	2 (3.5)	2.00 ± 1.41	1.00 ± 0.00	1.00 ± 1.41		0.71
**Musculoskeletal pain**						
Neck pain	20 (35.1)	4.20 ± 1.74	1.90 ± 1.62	2.30 ± 2.08	<0.001	1.11
Lower back pain	11 (19.3)	4.27 ± 1.56	2.00 ± 1.79	2.27 ± 2.05	0.004	1.11
Lower limb pain	9 (15.8)	4.11 ± 1.36	2.33 ± 2.06	1.78 ± 1.30	0.003	1.37
Upper limb pain	9 (15.8)	2.78 ± 1.48	1.00 ± 0.00	1.78 ± 1.48	0.007	1.20
Pain at other sites	7 (12.3)	4.43 ± 1.72	1.57 ± 1.51	2.86 ± 1.77	0.005	1.62
**Neurological symptoms (nervous system)**						
Headache	14 (24.6)	4.00 ± 1.57	1.00 ± 0.00	3.00 ± 1.57	<0.001	1.91
Dizziness	11 (19.3)	3.27 ± 1.74	1.36 ± 0.92	1.91 ± 1.81	0.006 †	1.06
Others	3 (5.3)	2.67 ± 1.53	2.67 ± 1.53	0 ± 3.00	1.000	0.00
**Urinary system**						
Nocturnal enuresis	10 (17.5)	3.60 ± 1.58	1.60 ± 1.07	2.00 ± 1.49	0.002	1.34
Frequent urination	8 (14.0)	3.88 ± 2.17	1.12 ± 0.35	2.75 ± 1.91	0.005	1.44
Diurnal enuresis	4 (7.0)	4.50 ± 3.00	2.25 ± 3.20	2.25 ± 2.63	0.186	0.86
Others	1 (1.8)	3.00 ± NA	1.00 ± NA	2.00 ± NA		

* *p*-value is calculated via the paired *t*-test. ** NRS: Numerical Rating Scale. † For variables that did not meet the normality condition, the Wilcoxon signed-rank test was also performed; the results are presented in Supplementary Table S1; NA: Not available.

**Table 4:** Results from the Pediatric Quality of Life Inventory (PedsQL™).

Scores by Dimensions	Mean ± SD	Summary Scores	Mean ± SD
**Ages 13–24 months** **The Parent Report for Infants of the PedsQL™ 1.0 Infant Scales (N = 1)**			
Physical functioning	91.67	Physical health	92.11
Physical symptoms	92.50		
Emotional functioning	64.58	Psychosocial health	80.77
School functioning	100.00		
Cognitive functioning	91.67		
**Total score**	85.56		
**Ages 2–4 years** **The Parent Report for Toddlers of the PedsQL™ 4.0 Generic Core Scales (N = 15)**			
Physical functioning	95.83 ± 8.73	Physical health	95.83 ± 8.44
Emotional functioning	87.00 ± 14.49	Psychosocial health	91.79 ± 10.14
Social functioning	94.67 ± 10.60		
School functioning	95.00 ± 8.21		
**Total score**	93.33 ± 9.55		
**Ages 5–6 years** **The Child and Parent Report for Young Children of the PedsQL™ 4.0 Generic Core Scales (N = 41)**			
Physical functioning	98.48 ± 3.90	Physical health	98.48 ± 3.9
Emotional functioning	94.27 ± 8.84	Psychosocial health	97.28 ± 4.58
Social functioning	99.02 ± 3.91		
School functioning	98.54 ± 3.40		
**Total score**	97.69 ± 4.11		

## Data Availability

All data in this study were extracted from publicly available academic literature. The data presented are available from the corresponding author upon reasonable request.

## Supplementary Materials

The following supporting information can be downloaded at: https://www.mdpi.com/article/10.3390/healthcare13151835/s1, Table S1: Variances from long-term follow-up survey which were tested by Wilcoxon signed rank test. Table S2: Patient Satisfaction Survey Results for Integrative Korean Medicine Treatment.

## Abbreviations

The following abbreviations are used in this manuscript:

TA	Traffic accident
KM	Korean medicine
WM	Western medicine
IKMT	Integrative Korean medicine treatment
EMR	Electronic medical record
NRS	Numeric Rating Scale
EQ-5D-5L	EuroQol-5 Dimension-5 Level
PedsQL™	Pediatric Quality of Life Inventory
AE	Adverse event
ROM	Range of motion
PMI	Permanent medical impairment
