# Supplementary material for: Safety and Effectiveness of Integrative Korean Medicine Treatment for Pediatric Patients After Traffic Accidents: Retrospective Chart Review and Survey Research with a Focus on Emotional and Behavioral Symptoms
Source: Healthcare (Basel). 2025 Jul 28;13(15):1835. doi: 10.3390/healthcare13151835 (PMC12345994; doi:10.3390/healthcare13151835)
Supplement: Supplementary file 1 [file healthcare-13-01835-s001.zip › healthcare-3640876-supplementary.pdf]

Supplemental Table S1. Variances from long-term follow-up survey which were tested by Wilcoxon signed rank test

Symptom	NRS (Mean ± SD)		P-value*
	Right After the TA	Present (at the Survey)	
**Anorexia**	3.00 (2.00-5.00)	1.00 (1.00-1.00)	<0.001
**Nausea, vomiting**	4.00 (3.00-5.00)	1.00 (1.00-1.00)	0.002
**Dizziness**	3.00 (2.50-4.00)	1.00 (1.00-1.00)	0.012
**Anxiety and avoidance**	5.00 (3.25-7.00)	1.00 (1.00-2.00)	<0.001

*P-value was calculated by Wilcoxon signed rank test

Supplemental Table S2. Patient Satisfaction Survey Results for Integrative Korean Medicine Treatment

**Overall satisfaction after treatment (N=57)**				
Very satisfied	Satisfied	Neutral	Dissatisfied	Very dissatisfied
29 (50.9%)	23 (40.4%)	4 (7.0%)	1 (1.8%)	0 (0.0%)

Satisfaction levels by treatment type

**Acupuncture treatment satisfaction (N=26)**		
Satisfied	Neutral	Dissatisfied
22 (84.6%)	4 (15.4%)	0 (0.0 %)

**Reasons for satisfaction with acupuncture (Multiple responses allowed) (N=22)**	
Pain improvement	10 (45.5%)
Improvement in anxiety and psychological symptoms	16 (72.7%)
Others	0 (0.0%)

**Cupping treatment satisfaction (N=3)**		
Satisfied	Neutral	Dissatisfied
3 (100%)	0 (0.0 %)	0 (0.0 %)

**Reasons for satisfaction with cupping (Multiple responses allowed) (N=3)**	
Pain improvement	2 (66.7%)
Improvement in anxiety and psychological symptoms	2 (66.7%)
Others	0 (0.0%)

**Herbal medicine treatment satisfaction (N=41)**		
Satisfied	Neutral	Dissatisfied
27 (65.9%)	10 (24.4%)	4 (9.8%)

**Reasons for satisfaction with herbal medicine treatment (Multiple responses allowed) (N=27)**	
Pain improvement	14 (51.9%)
Improvement in anxiety and psychological symptoms	23 (85.2%)
Others	2 (7.4%)

**Reasons for Dissatisfaction with Herbal Medicine Treatment (Multiple Responses Allowed (N=4)**	
Aversion to herbal medicine	0 (0.0%)
Discomfort with the taste of herbal medicine	4 (100.0%)
Side effects after taking herbal medicine	0 (0.0%)

* The data are presented as N (percentage).
